# The optimal formulation of a readily compostable horticultural growing substrate for vertical farming was determined using design of experiments

**DOI:** 10.1038/s41598-024-80650-5

**Published:** 2024-11-25

**Authors:** Jonathan M. Romo, Isabel N. Smith, Michael Galloway, Timothy J. Cuthbertson

**Affiliations:** 1 VelociGro, Inc., Monrovia, CA USA; 2 Control Laboratories, Inc., Watsonville, CA USA

**Keywords:** Gels and hydrogels, Field trials, Environmental impact

## Abstract

A novel, optimized, polysaccharide and biochar-based, compostable hydrogel horticultural growing substrate for use in hydroponics and vertical farming was created based upon empirical methods and statistical design of experiments. A 15-run D-optimal mixture design of experiments was completed that increased the 14-day plant growing ability of a five-component hydrogel nearly ten-fold from 4.3695 g to 41.2623 g per 100 plants. The data were analyzed using a standard least squares method with an effect screening emphasis, and a model was created that maximized the signal to noise ratio. There was a good correlation between the measured and predicted values of the model, with an r-squared value of 0.90. The predictions of efficacy and compostability were confirmed with subsequent experiments that showed the hydrogel was composted in less than 84 days and that the plant growth predicted by the model differed from the experimental growth by 0.65%. The resulting optimized formulation had a high fertilizer content for a growth medium. We therefore suggest that an empirical approach to formulation research can produce superior outcomes with a statistically designed study.

Plant based foods can be grown outdoors on naturally irrigated land, outdoors on artificially irrigated land, indoors using artificial irrigation and natural light (for instance, in a greenhouse or hoop farm), or indoors using both artificial irrigation and light (in a vertical farm)^1^. All these approaches have their advantages and disadvantages. However, vertical farming is becoming increasingly economically viable as Earth’s population and average temperatures continue to increase^2^. Vertical farming uses far less water, provides more optimal conditions for plants to grow, and uses less land than any other farming technique^3^. Therefore, there is increasing interest in creating vertical farms near population centers^4^. However, while much attention has been paid to optimizing the growing conditions of vertical farms by controlling the atmosphere^5^, photosynthetic active radiation (PAR)^6^, and hydroponic solution attributes^7^, far less attention has been paid to optimizing the horticultural growing substrates^8^. The state of the art in horticultural growing substrates is dominated by rockwool^9^, peat^10^, and coconut coir^11^. Rockwool is made of mined materials that are neither biodegradable nor compostable^12^. Peat is harvested from the environment, and while it is compostable, it is not readily renewable (as peat can take 1000 years to regenerate naturally)^13^. Coconut coir is also compostable but can transfer pests, bacteria, and fungi to vertical farms^14^. The ideal substrate would contain renewable ingredients, be readily compostable, pest-free, and grow plants well^15^. We report here the development of seaweed-based^16^, compostable, pest-free substrate that grows plants well.

We endeavored to find a plant-based gelling agent that was beneficial for crops, compostable^17^, easily renewable^18^, and with enough structural integrity to work as a horticultural growing substrate for one to two months.^19^ Various candidates were considered, including agar^20^, gellan gum^21^, chitosan^22^ and carrageenan^23^. K-carrageenan emerged as the best candidate because it had strong gelling characteristics^24^ and was a known plant growth promoter^25^. Furthermore, as many fertilizers are cationic^26^, and k-carrageenan gel strength was increased in the presence of cross-linking cations like calcium^27^ there was a good overlap in function.

The other ingredients studied were fertilizers, a buffer, and a biochar purification agent. Murashige & Skoog (M&S) plant growth medium was selected as the fertilizer because it was known to support the growth of a wide variety of crops^28^. Calcium citrate was chosen as the buffer because it was known to be compatible with M&S fertilizers^29^. Finally, activated charcoal derived from biochar wood was designated as the purification agent because it was also a known plant growth promoter^30^.

Optimization of a five-component hydrogel by changing one variable at a time (OVAT) would have been at best inefficient and at worst would have failed to find the optimal formulation^31^. Conversely, design of experiments has emerged as a technique that maximizes the value of research and produces more robust formulations^32^. We found that by varying the concentrations of ingredients with wide ranges we could find optimized formulations with unusual properties. Of course, as we were conducting mixture design of experiments, all the components were restricted to adding up to 100%.

As the goal of this research was to produce a growing medium useful for today’s vertical farms, the design of experiments was tailored to maximize the growth of crops common to them^33^. The state of the art in vertical farming varies widely from company to company and there are many watering systems that are employed. Some of the most common hydroponics systems are ebb and flow, nutrient film technique (NFT), vertical drip tower, and deep-water culture^34^. We focused solely on the initial 14-day propagation stage of plant growth, during which no watering systems were used, to enhance the universality of our results. Plants that are larger at 14 days are more likely to grow faster during the hydroponics production phase due to their increased leaf surface area^35^.

Likewise, the conditions chosen for the plant propagation study were chosen to be consistent with best practices: humidity domes for germination^36^, reverse osmosis (RO) water^37^, a commonly used fertigation solution^38^, and LED lighting with appropriate cycling^39^.

Therefore, a five-component formulation of established plant growth promoters was created for cultivars commonly grown in vertical farms using hydroponics best practices. We were able to optimize the formulation using only 15 experiments because of the efficiency of design of experiments. The purpose of this paper is to serve as a model for the rapid research and development of plant growing media.

## Materials and methods

### Statistical design of experiments

The formulation was optimized to enhance leafy green growth across 15 experiments, each involving 100 plants (15 half-trays of 200 × 4.4 cm grow plugs). Statistical design of experiments was conducted using JMP software (licensed from JMP Statistical Discovery LLC.) to evaluate the simultaneous effects of multiple formulation components and to ensure product quality and process capability. A mixture design was chosen to ensure that the ingredients totaled 100%. JMP recommended a 15-run study with a D-optimal design (D efficiency = 0.003277), which was suitable for this application as it allowed for a cost-effective study that could serve as a template for future product designs. Additionally, the D-optimal design was ideal due to the tightly constrained parameters of the mixture. The results of the 15 runs were compared against a previously developed hydrogel formulation and a coconut coir control.

### Raw materials

All raw materials were purchased as food grade. Purified water was generated in-house by treating municipal water by reverse osmosis just as one would do at a vertical farm. Refined k-carrageenan was purchased from W Hydrocolloids. Acidic biochar, M&S basal medium, and calcium citrate were purchased from Sigma-Aldrich.

### Plant substrate formulation

The horticultural substrates were made in 2 L batches by adding the proportions listed in Table 1 by mass. RO water was added to a 3 L beaker on a hotplate and overhead stirring was initiated at 400 RPM. The solid ingredients were mixed by hand in a separate beaker and then added portion wise to avoid agglomeration. Heating was initiated on the hotplate to warm the mixture to 85 ºC. When the mixture was substantially homogeneous, stirring was slowed to 100 rpm. The mixture was maintained at 85 +/- 3 ºC for 30 min, at which time the heat was turned off and the mixture allowed to cool to less than 60 ºC. The mixture was poured into a 200 × 4.4 cm tray that had the bottom holes plugged with a silicone mat. The plugs were allowed to cool to room temperature and then they were dibbled with a pen in preparation for seeding.

**Table 1:** Comparison of the compositions and plant growth performance in all cultivars combined in the 15-run design of experiments formulations to the gel control coir control and optimized formulation (predicted) and average of the confirmation run.

Run	Water (mass fraction)	K-carrageenan (mass fraction)	Biochar (mass fraction)	M&S (mass fraction)	Calcium Citrate (mass fraction	14-day total plant mass (g)
1	0.98100	0.01000	0.00100	0.00400	0.00400	1.6486
2	0.98000	0.01700	0.00100	0.00200	0.00000	25.3899
3	0.98000	0.01000	0.00400	0.00200	0.00400	13.9062
4	0.98200	0.01000	0.00400	0.00400	0.00000	35.9626
5	0.98100	0.01000	0.00100	0.00400	0.00400	1.5914
6	0.98700	0.01000	0.00100	0.00200	0.00000	22.4868
7	0.98000	0.01100	0.00100	0.00400	0.00400	1.5302
8	0.98000	0.01000	0.00400	0.00200	0.00400	10.5598
9	0.98000	0.01200	0.00400	0.00400	0.00000	30.9997
10	0.98000	0.01700	0.00100	0.00200	0.00000	26.0163
11	0.98700	0.01000	0.00100	0.00200	0.00000	35.0747
12	0.98700	0.01000	0.00100	0.00200	0.00000	35.9909
13	0.98000	0.01000	0.00400	0.00200	0.00400	19.5847
14	0.98200	0.01000	0.00400	0.00400	0.00000	43.8156
15	0.98000	0.01700	0.00100	0.00200	0.00000	18.3488
Gel control	0.97925	0.01500	0.00300	0.00275	0.00000	4.3695
Coir control	N/A	N/A	N/A	N/A	N/A	28.2394
**Optimized (predicted)**	**0.98400**	**0.01000**	**0.00400**	**0.00200**	**0.0000**	**40.9936**
**Confirmation of optimized**	**0.98400**	**0.01000**	**0.00400**	**0.00200**	**0.0000**	**41.2623**

### Electrical conductivity and pH measurement

The Electrical Conductivity (EC) and pH of prepared gels were measured by placing an EC/pH probe (Extech EC-500) inside a gel plug and waiting for 30 s or until the measured value stabilized.

### Plant growth studies

**Fig. 1:**
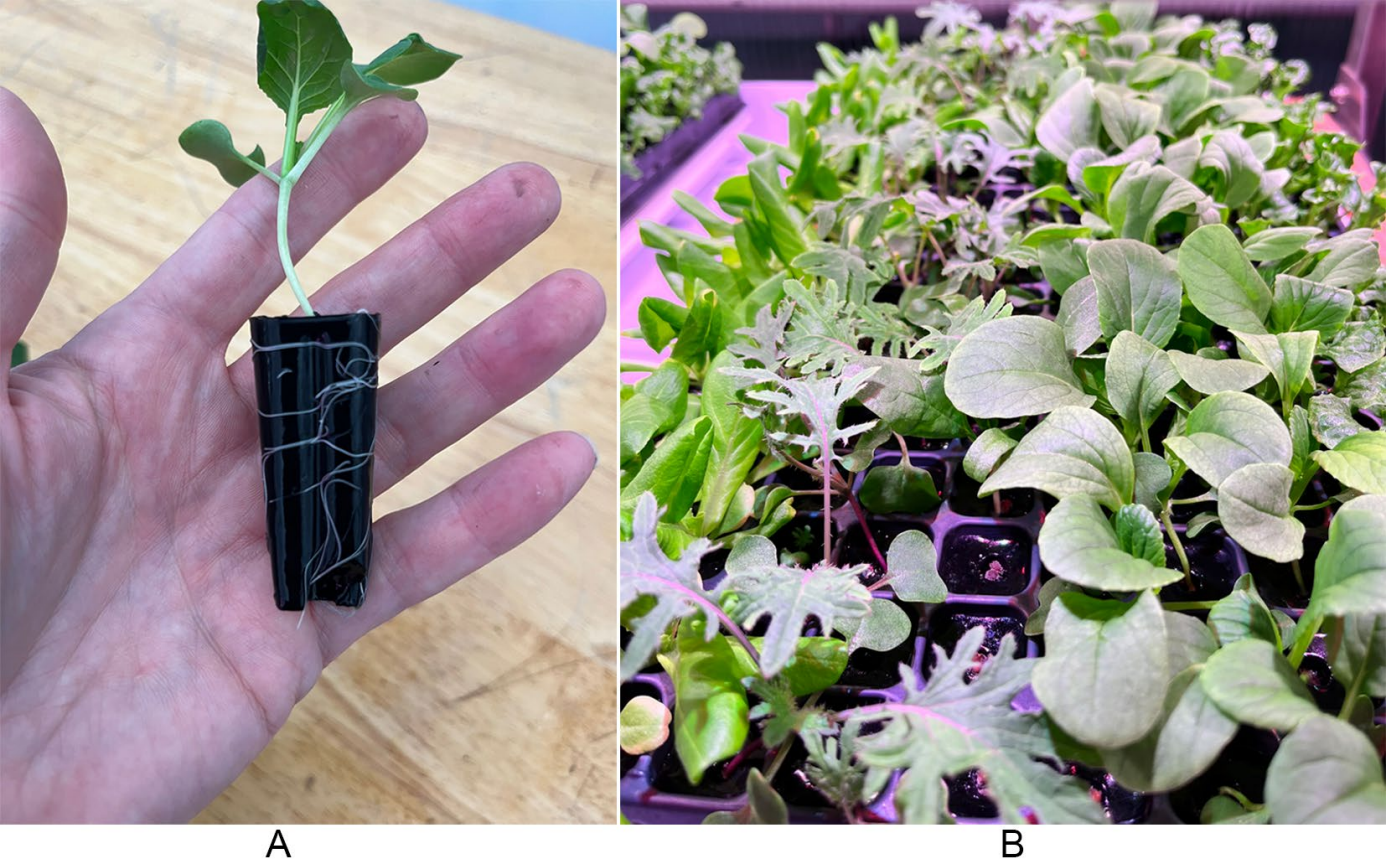
Bok choy growing in the compostable hydrogel substrate (left) and the plants growing from the substrate in a 200 × 4.4 cm tray (right).

The performance of the formulations was evaluated for the growth and health of five cultivars (*N* = 1500): fairly butter lettuce and F1 crystal lettuce (purchased from Paramount Seeds Company), and bok choy, spinach, and kale (purchased from Johnny’s Selected Seeds). 200 × 4.4 cm trays and the appropriate humidity domes were purchased from T.O. Plastics (Fig. 1). During germination the plugs were placed under a humidity dome and spritzed with fertigation solution daily with an EC of 0.3 mS/cm. Lighting in the vertical nursery carts was set to 250 PAR and with a 20/4-hour day/night cycle. Climate in the nursery carts ranged from 18 to 28 °C with a relative humidity of 50–90%. After seven days, the humidity domes were removed. At 14 days the plants were cut at crown level, separated by cultivars, and immediately weighed for wet mass.

### Confirmation run of the model’s predicted optimized formulation

One 100 kg confirmation batch of the optimized formulation recommended by the design of experiments model was completed and a portion of it was tested for plant growth. The EC and pH of the batch were measured to be 4.26 mS/cm and 6.99, respectively. Three side-by-side trials of 20 plants of each cultivar (tray size 200 × 4.4 cm) were subjected to the same plant growth testing as completed in the design of experiments.

### Compostability study

Compostability was measured per ISO 20200 (lab scale disintegration). The composting vessels were 5 L in size (36 cm X 20 cm X 11 cm) and were made from non-degradable plastic. The vessels’ lids had six latches and a gasket to ensure a tight seal. Per the method, for aeration a 5 mm diameter hole was drilled on either end of the container, approximately 6.5 cm from the bottom. The containers were incubated in an incubator that kept the temperature at 58 +/- 2 ºC throughout the 84 days of composting.

A synthetic compost inoculum was prepared using rabbit feed, mature compost, corn starch, saccharose, corn seed oil, urea, sawdust, and wood chips in the following mass proportions: 30%, 10%, 10%, 5%, 4%, 1%, and 40%. This was thoroughly mixed and then sieved through a 9.5 mm sieve. The urea content was adjusted to achieve a carbon-to-nitrogen ratio of 28:1 to 32:1, and water was added to reach a moisture content of 55% by weight. The mature compost was a 3-month-old mixture from the Monterey District composting facility.

In each vessel, 1 kg of the inoculum was combined with 500 g of whole test sample plugs, ensuring even distribution of the compost. The mixture was composted for 84 days at a controlled temperature of 58 ± 2 ºC, following the ISO 20200 procedure outlined in Table 2.^40^ Observations were recorded periodically. After 84 days, the material was removed from the vessels and screened through a 2 mm sieve. The test was deemed successful if no more than 10% of the original dry weight remained on the sieve. The compost produced from these disintegration studies was then mixed with control compost for ecotoxicity quality testing.

**Table 2:** The ISO 20200 list of reactor control and measurement used for the compostability study.

Time (days)	Operation
0	Initial mass of reactor was recorded
1,2,3,4,7,9,11,14	Reactor was weighed and water was added to restore the initial mass. The reactor was mixed.
8,10,16,18,21,23,25,28	Reactor was weighed and water was added to restore the initial mass. The reactor was not mixed.
30–60	Reactor was weighed and water was added to restore the mass to 80% of the initial mass. The reactor was mixed.
60–84	Reactor was weighed and water was added to restore the mass to 70% of the initial mass. The reactor was mixed.

### Quality of compost test (ecotoxicity)

The quality of compost produced from the plant growing substrate was compared to mature compost as a control following the OECD Guideline 208 with the modifications found in Annex E of EN 13432^41^. In brief, to pots with clear plastic covers were added the compost mixtures to be studied (25% and 50% by weight test compost mixed with mature compost). Two cultivars of plants were used for the study, barley, and cucumber. 100 cucumber seeds were planted in each dilution. 50 barley seeds were planted in each dilution. Triplicates of each dilution were made. Percent germination was determined using percent germination compared to the control as 100%. The dry weight of the plants was used to determine biomass.

Heavy metals analyses were conducted using established methods: As, Cd, Cu, Pb, Ni, Se, Zn, Co, Cr, and Mo digestion using EPA 3050B (Analysis EPA Method 6020, ICPMS);^42^ and Hg using EPA 7471 (Cold Vapor)^43^. Total fluorine was analyzed according to EN 14582, using combustion in an oxygen bomb, trapping the gases in DI water, and then measuring fluoride by ion selective electrode^44^. EN 14582 allows for several possible technologies to be used for the final detection of the trapped fluoride and states the determinative method used must be referenced. The method used for determining fluoride was ASTM D1179-16 (reapproved in 2021)^45^. The combustion step converts fluorine compounds to fluoride, therefore giving the total fluorine concentration.

## Results

### The formulation components effects on plant growth

**Fig. 2:**
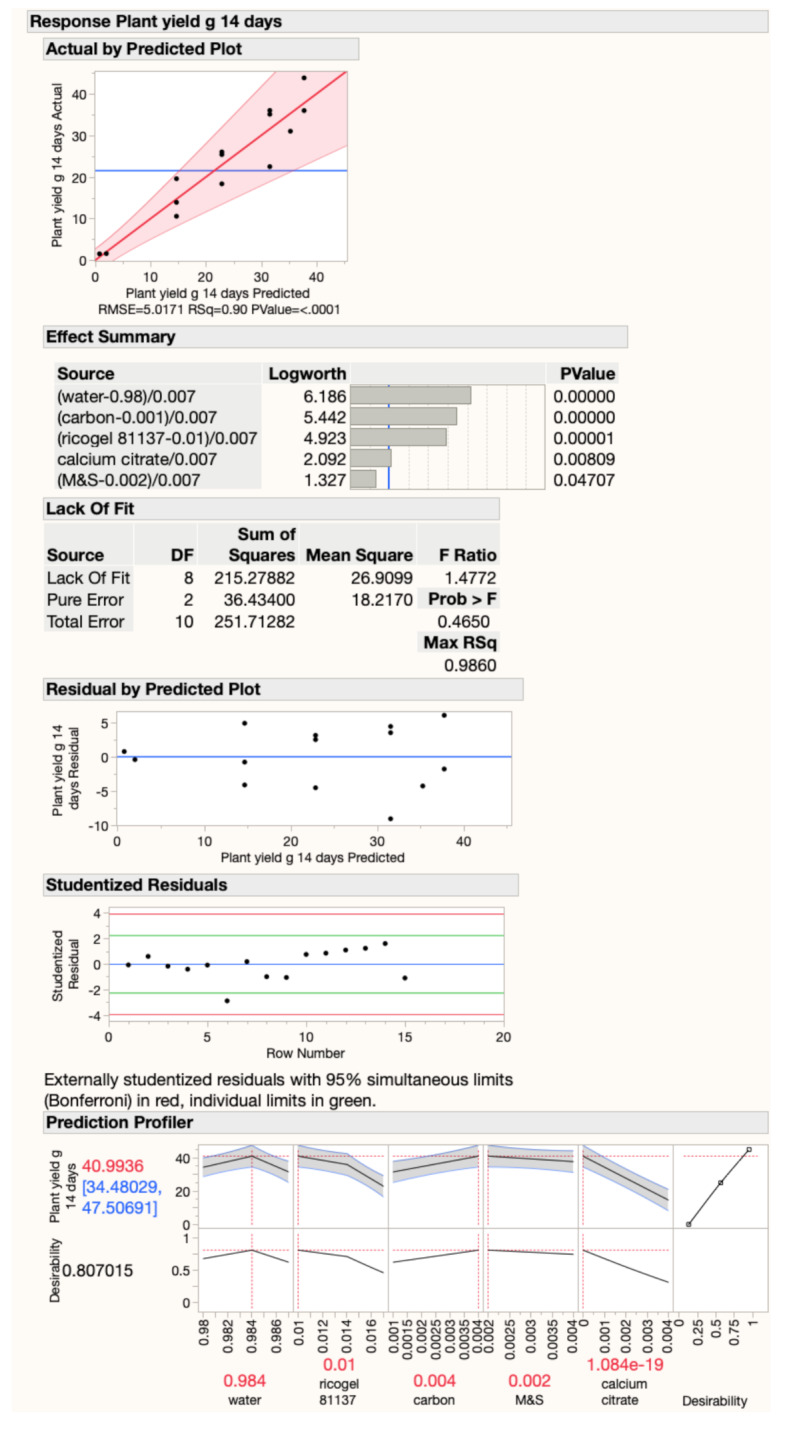
Results of the JMP model fit, R^2^ = 0.90. The effect summary indicates that all five formulation ingredients are likely to be significant. The Lack of Fit, Residual by Predicted Plot, and Studentized Residuals are indicative of a strong model with no trial results statistically excludable. The Prediction Profiler was used to maximize predicted plant yield at 14 days, resulting in the formulation with the highest desirability.

After the 14-day wet masses of the plants cut at crown level were measured in the plant growth studies (Table 3), these masses were combined and JMP was used to fit a model using standard least squares analysis with an emphasis on effect screening (Fig. 2). The model effects were each of individual formulation components crossed with the mixture. The least squares fit found that all the individual formulation components likely had a significant effect on plant growth with > 95% confidence. The actual versus predicted plot for plant mass had an R^2^ = 0.90. The order of ingredient importance was water > biochar > carrageenan > calcium citrate > M&S with log Worth values of 6.2, 5.4, 4.9, 2.1, and 1.3, respectively (log Worth is -log of P value). The JMP prediction profiler used the model to maximize predicted plant mass. The model’s predicted optimized formulation is included in Table 1 along with the control formulations and the 15 design of experiments runs.

**Table 3:** 14-day wet masses of plants cut at crown level. The optimized formulation produced nearly ten times more plant mass compared to the previous gel control.

Run	Butter Lettuce (g)	Bok Choy (g)	Spinach (g)	Kale (g)	Crunchy Lettuce (g)	Total (g)
1	0.0359	0.8423	0.7704	0.0000	0.0000	1.6486
2	2.0622	12.5825	5.3342	4.7441	0.6669	25.3899
3	0.3481	7.0026	3.5822	2.2625	0.7108	13.9062
4	5.0129	15.7790	5.3492	7.7811	2.0404	35.9626
5	0.0000	0.5614	1.0300	0.0000	0.0000	1.5914
6	2.3894	11.6894	0.5907	7.3260	0.4913	22.4868
7	0.1591	0.8300	0.4479	0.0878	0.0064	1.5302
8	1.2974	6.6863	0.2267	1.6577	0.6917	10.5598
9	6.3073	14.5016	4.4080	3.7866	1.9962	30.9997
10	2.9445	15.3108	4.7711	1.8465	1.1434	26.0163
11	5.6092	18.3187	2.7729	7.1258	1.2481	35.0747
12	3.7128	21.4757	1.1400	8.1048	1.5576	35.9909
13	3.4924	10.1125	2.3608	3.0067	0.6123	19.5847
14	9.5061	18.6366	5.5509	7.5365	2.5855	43.8156
15	2.4320	10.9538	2.1682	1.5545	1.2403	18.3488
**Gel Control**	**0.0798**	**1.6851**	**1.9204**	**0.6431**	**0.0411**	**4.3695**
Coir Control	5.1080	10.5716	5.0094	5.2410	2.3094	28.2394
**Confirmation of Optimized**	**11.8750**	**14.3498**	**2.3262**	**6.9131**	**6.3982**	**41.2623**

### Confirmation of optimized formulation study

The average plant mass per tray was found to be 41.2623 g (SD = 1.4016 g) whereas the statistical model predicted 40.9936 g of plant mass per tray (a percent error of 0.65%).

### Compostability study

The plant growing substrate was found to be 100% disintegrated after the test compost was passed through a 2 mm sieve per ISO 20200.

### Quality of compost study (ecotoxicity)

The plant growing substrate was found to have no significant effect on the germination rate nor the biomass versus control for barley and cucumber (Table 4).

**Table 4:** The effect of test compost on germination and biomass in the growth of barley and cucumber.

Species	% Test compost by weight	Germination (% of control)					Biomass (% of control)				
		Trial			Mean	s	Trial			Mean	s
		1	2	3			1	2	3		
Barley	25	97.2	99.3	97.2	97.9	1.2	89.7	93.2	93.2	92.0	2.0
Barley	50	97.8	100.0	104.3	100.7	3.3	93.0	95.9	111.8	100.2	10.1
Cucumber	25	99.3	100.3	99.3	99.7	0.6	97.5	99.8	100.3	99.2	1.5
Cucumber	50	101.7	96.5	100.7	98.9	6.9	103.3	91.0	102.5	98.9	6.9

No heavy metals were detected in the growth medium except for Zn, resulting in the material meeting the Pass/Fail standards in US, Canada, Europe, and Japan. No fluorine was found in the plant substrate, and the material met the criteria for pass/fail set at 100 mg/kg (table 5).

**Table 5:** Results of heavy metals and fluorine analysis of the plant growing substrate. “ND” stands for “not detected” and “NA” indicates that that region has no Pass/Fail standard.

Analyte	Results (mg/kg)	Reporting Limit (mg/kg)	Pass or Fail of Standards by Region			
			US	Canada	Europe	Japan
As	ND	0.5	Pass	Pass	Pass	Pass
Cd	ND	0.5	Pass	Pass	Pass	Pass
Cu	ND	0.5	Pass	NA	Pass	Pass
Pb	ND	0.5	Pass	Pass	Pass	Pass
Hg	ND	0.2	Pass	Pass	Pass	Pass
Ni	ND	0.5	Pass	Pass	Pass	Pass
Se	ND	0.5	Pass	Pass	Pass	NA
Zn	1.2	0.5	Pass	Pass	Pass	Pass
Co	ND	0.5	NA	Pass	NA	NA
Cr	ND	0.5	Pass	NA	Pass	Pass
Mo	ND	0.5	NA	Pass	Pass	NA
F	ND	20	Pass	Pass	Pass	Pass

## Discussion

The purpose of this study was to determine whether a mixture design of experiments could identify the optimal formulation for a five-component, compostable, plant-based growing substrate suitable for hydroponic systems and vertical farms. A 15-run D-optimal study was developed using the JMP Custom Designer software platform. This approach was recommended by JMP and was appropriate given that the formulation components needed to total 100%, along with specific constraints on ingredient percentages. Excessive polymer content would result in a mixture that could not be stirred or poured, while high fertilizer concentrations could create a mixture too salty for plants. Similarly, too much calcium citrate would lower the pH beyond suitable levels for plant growth, and an excessive amount of biochar could compromise the substrate’s structural integrity. Although other design optimalities, such as A- and G-optimal designs, could have been employed, D-optimal design tends to yield superior results for highly constrained mixtures while minimizing resource allocation^46^.

The D-optimal mixture design recommended by JMP minimized the variance of the constrained design space by distributing the prescribed mixture fractions of the five ingredients throughout the design space. Of course, the variance is always lowest near the center of the design space, and one could decrease the variance near the extremes of the design space by adding tested ingredient fractions outside the design space via design augmentation. We should comment that the fractions recommended by the model are at the extremes of the design space for four out of the five mixture components (k-carrageenan, biochar, M&S, and calcium citrate) and therefore further refining of this model is desirable before finalization of this formulation. However, it should be noted that there was a strong agreement between the predicted plant growth of the optimized formulation by the model and the average plant growth of the test batch, with percent error < 1%. This was impressive considering the tests could not be run simultaneously and because plant growth over a 14-day period was necessarily variable. Moreover, Run 14 (Table 1), was like the optimized formulation and demonstrated a similar level of plant growth in 14 days. Had the confirmation batch produced a substantially different plant growth to the model prediction then design augmentation would have been even more appropriate.

The design of experiments test batches were compared with a gel control, which was the original gel substrate formulation that this study was attempting to improve. One can see from Table 1 that the plant mass at 14 days was increased nearly ten-fold from 4.3695 g to 41.2623 g per 100 plants. This demonstrated how sub-optimal the original gel substrate formulation was. It also showed that with only 15 test batches substantial refining of a formulation was possible using a design of experiments mixture design.

The confirmation batch had a relatively high EC of 4.26, which was about twice the EC of a typical hydroponics solution^47^. This result supports the theory that effective mixture design of experiments models rely upon purposely exiting the first principles regime and moving instead toward an empirical approach^48^. In other words, the purpose of a study is to maximize, minimize, or match the target value of a chosen experiment response without regard to the reasoning behind the result. An empirical approach to design of experiments was therefore more likely to be successful^49^.

Among the 15 test batches in the design of experiments study there was significant variation in the growth of the five cultivars. This reinforces the notion that different cultivars may flourish in different formulations, and that formulations can be tailored to maximize the growth of specific cultivars^50^. This presents a significant opportunity in indoor farming, as it allows for the cultivation of cultivars that struggle in traditional outdoor settings or are not suitable for shipping. For example, many cultivars cultivated outdoors were selected for their frost hardiness, pest resistance, and ease of transport^51^. Nevertheless, many cultivars that were selected for outdoor farms are currently being grown in indoor farms^52^. It is reasonable to expect that indoor farms near urban centers can enhance the cultivation of fruits and vegetables with superior culinary qualities. These farms benefit from reduced shipping distances, controlled growth parameters, minimized pesticide use, and the ability to design substrates that optimize growth and flavor. We believe that our research can exploit the natural variability observed among cultivars to make these designed substrates.

Hydrogels are well known in tissue culture applications^53^, but finding a formula that can withstand the watering systems used in a vertical farm, breaks down in a composting facility, and grows plants well is more challenging. Design of experiments has been used to optimize the growing conditions in vertical farms, however often these studies have been designed around growth parameters rather than substrates^54^.

We demonstrated the feasibility of creating a stable hydroponic plant growing substrate that is also readily compostable. The results from biodegradability testing aligned with our predictions; in the disintegration test, the only component of the substrate that needed to break down was the crosslinked k-carrageenan, a polysaccharide derived from seaweed. Thus, it is reasonable to expect that a compost mixture containing microorganisms would effectively disintegrate this hydrogel.

The quality of compost study (ecotoxicity) result was consistent with our predictions, because k-carrageenan is used as a plant growth stimulator, M&S is an established fertilizer, and biochar is known to be compatible with a wide variety of plants. In the barley with 25% test compost there was a slight decrease in the rate of percent germination versus control and percent biomass versus control (97.9% and 92.0%, respectively), however this effect was not dose-dependent because when 50% test compost was used with the barley the percent germination and percent biomass were like control soil (Table 4). We believe there is no scientific reason that 25% test compost would be toxic to barley when 50% test compost was not toxic to barley. The hydrogel plant growing substrate met the criteria for passing the ecotoxicity test outlined in OECD Guideline 208.

In addition to producing high-quality compost, the optimized formulation excludes environmentally harmful ingredients. Our analysis of heavy metals and fluorine confirmed our predictions, as the specifications for the food-grade carrageenan and biochar used indicated minimal levels of these substances (Table 5). Although M&S contains trace amounts of Zn, Cu, and Mn, these concentrations are not significant. Heavy metals can pose serious risks to the environment and are a persistent issue in industrial composting facilities^55^.

While indoor farms can be more environmentally friendly than traditional outdoor farms, they still pose significant environmental challenges^56^. These farms often require substantial electricity for lighting and pumps, which may be produced through carbon-intensive methods. Additionally, they frequently rely on single-use plastics for plant containers and final products, and they employ synthetic chemicals. Furthermore, the plant growing substrates used are often not sustainably sourced and are typically sent to landfills after use. We hope our research will contribute to improving the sustainability of these substrates.

## Conclusion

A five-component mixture was rapidly optimized using a 15-experiment design of experiments created with JMP software. The substrate developed in this study consisted of biocompatible and compostable ingredients, ensuring that the final mixture was also biocompatible and compostable, as no chemical reactions occurred during processing except for the gelling of the k-carrageenan polysaccharide. The optimized substrate demonstrated nearly ten times the plant growth rate of the previous gel formulation using the same ingredients, and compostability studies confirmed its rapid disintegration without toxicity to plants. Chemical analyses revealed the absence of heavy metals and fluorine compounds, which can be harmful to the environment. Future studies should investigate growth variations among different cultivars to develop custom formulations that support fragile or difficult-to-grow plants in controlled indoor farm conditions. Since the optimized formulation component fractions were near the edges of the design space for four out of the five ingredients, further augmentation of the design of experiments is recommended before finalizing the formulation.

## Data Availability

The datasets generated during the current study are available from the corresponding author on reasonable request.
